# Multiscale deformations lead to high toughness and circularly polarized emission in helical nacre-like fibres

**DOI:** 10.1038/ncomms10701

**Published:** 2016-02-24

**Authors:** Jia Zhang, Wenchun Feng, Huangxi Zhang, Zhenlong Wang, Heather A. Calcaterra, Bongjun Yeom, Ping An Hu, Nicholas A. Kotov

**Affiliations:** 1 Key Laboratory of Micro-systems and Micro-structures Manufacturing, Ministry of Education, Harbin Institute of Technology, Harbin 150080, China; 2 Department of Chemical Engineering, University of Michigan, Ann Arbor, Michigan 48109-2136, USA

## Abstract

Nacre-like composites have been investigated typically in the form of coatings or free-standing sheets. They demonstrated remarkable mechanical properties and are used as ultrastrong materials but macroscale fibres with nacre-like organization can improve mechanical properties even further. The fiber form or nacre can, simplify manufacturing and offer new functional properties unknown yet for other forms of biomimetic materials. Here we demonstrate that nacre-like fibres can be produced by shear-induced self-assembly of nanoplatelets. The synergy between two structural motifs—nanoscale brick-and-mortar stacking of platelets and microscale twisting of the fibres—gives rise to high stretchability (>400%) and gravimetric toughness (640 J g^−1^). These unique mechanical properties originate from the multiscale deformation regime involving solid-state self-organization processes that lead to efficient energy dissipation. Incorporating luminescent CdTe nanowires into these fibres imparts the new property of mechanically tunable circularly polarized luminescence. The nacre-like fibres open a novel technological space for optomechanics of biomimetic composites, while their continuous spinning methodology makes scalable production realistic.

## Abstract (web summary)

The mechanical properties and hierarchical structure of nacre have been widely investigated as a biomimetic template for applications. Here, the authors demonstrate that nacre-like fibres made from nanoplatelets and polymers show exceptional stretchability and toughness.

## Graphical abstract

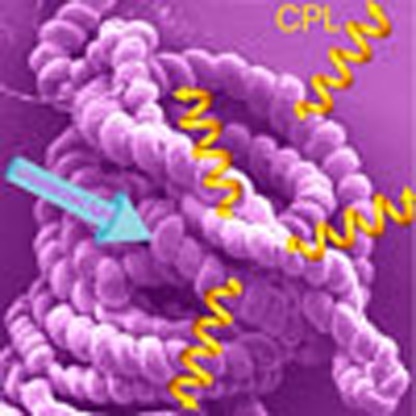

Realization of materials with high toughness combined with other properties is one of the key challenges for both load-bearing and functional materials^1^. This materials science challenge can often be addressed using biomimetic design taking naturally occurring composites that have been optimized over long evolutionary periods as inspiration. The ‘brick–and–mortar' layered design of nacre, with alternating layers of inorganic platelets and biopolymers, inspired biomimetic research for several decades^2^,^3^. The materials architecture with alternating layers of hard inorganic components and soft organic polymers effectively arrests the propagation of cracks. The process has been replicated using a large variety of inorganic components, including clay^4^,^5^,^6^,^7^, Al_2_O_3_ (refs 1, 8) and layered double hydroxides^5^, combined with various organic polymers including poly(vinyl alcohol) (PVA)^5^, polyelectrolytes^6^ and chitosan^7^. The layered biomimetic nanomaterials are much tougher than their inorganic and organic components alone, and often reveal exceptionally high strength and stiffness^1^,^9^. Further improvement of toughness in biomimetic nanocomposites is restricted, however, by the low strains (*ɛ*) of composite materials, especially when the volume fraction of the stiff inorganic phase is high. The nacre-like composites typically show strains <5% (ref. 10). In fact, the problem of low stretchability is quite general and observed for a variety of nanocomposites including graphene ribbons (*ɛ*=6%)^11^.

The combination of two structural motifs at different scales, specifically nanoscale and microscale in this case, is designed to simultaneously increase both the stretchability and toughness of a composite^12^,^13^. Indeed, a stretchable graphene film combined with ripples and yarns exhibited improved tensile strain of 30% (ref. 12) and 76% (ref. 13), respectively. This inspired our search for methods to create nacre-like composites with multiscale structural motifs and evaluate their mechanical properties, which we expected to be quite unique as well as technologically valuable. Here we demonstrate that it is possible to transform flat nacre films into fibres that combine layered nanoscale and spiral microscale structural motifs. The resulting fibres can sustain longitudinal strains as high as 414%. This is 10–1,000 times higher than typical biomimeticaly designed layered composites and other fibre-like nanocomposites. The nacre-like fibres display an unusually high gravimetric toughness of ∼640 J g^−1^, which significantly exceeds those of natural nacre (∼1 J g^−1^)^9^, dragline silk (165 J g^−1^)^14^, graphene (17 J m^−3^)^13^, Kevlar(KM2) (78 J g^−1^)^14^ and some of the best examples of composited single-wall carbon nanotube (SWNT) fibres (570–970 J g^−1^)^15^,^16^,^17^. Such unusual mechanical properties are attributed to multiscale deformation combining both the sliding of nanoscale platelets and unravelling of microscale spiral curls. Moreover, the described process of fibre spinning and strain-induced particle self-organization enables continuous scalable production of this material^18^,^19^. Furthermore, the helical patterns of the multiscale deformations causes circularly polarized luminescence (CPL) to be emitted at ∼575 nm when cadmium telluride (CdTe) nanowires are incorporated into PVA/CaCO_3_ fibres. The high stretchability of the fibres allows the wide-range modulation of the luminescence dissymmetry ratio (*g*_lum_) and the same is expected for many other optically active materials and different wavelengths. This novel optomechanical property of the fibres highlights the emergence of novel possibilities for engineering chiral nanomaterials that may be useful for remote monitoring of materials' strains.

## Results

### Preparation of CaCO_3_ and graphene nanoplatelets

To prepare the nacre-like fibres we used two types of inorganic ‘building blocks': one is platelets of CaCO_3_ (vaterite) synthesized from calcium chloride and ethylene glycol by a hydrothermal method, and the other is graphene-based nanosheets (*G*) made by electrochemical exfoliation of highly oriented pyrolitic graphite. The vaterite plateletes had diameters and thicknesses of 4–20 μm and 100–500 nm (Supplementary Fig. 1), respectively; these dimensions are very similar to the microplatelets of the aragonite inorganic phase in seashell nacre^9^. *G* nanosheets displayed diameters and thicknesses of 5–45 μm and 1–5 nm, respectively (Supplementary Fig. 2a–c). CaCO_3_ and *G* were chosen a pair of building blocks for the nacre-like composites because of their low density of defects^18^, (Supplementary Fig. 2d,e) and they are known to display tensile strength and stiffness higher than the two-dimensional (2D) nanocarbon materials prepared from other oxidation methods, such as Hummers' method. For the polymeric component in both types of nanocomposites we used PVA, which exhibits affinity to, and forms stable dispersions with, both CaCO_3_ and *G*^20^,^21^.

### Spinning of nacre-like fibres

**Figure 1:**
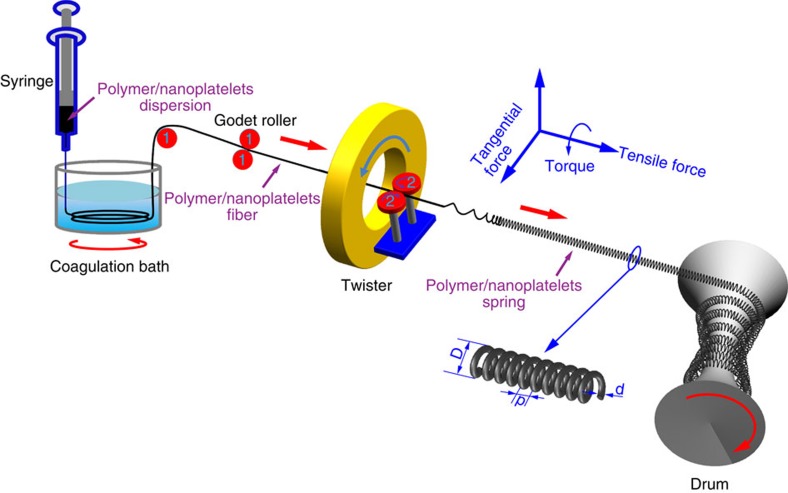
Schematic diagram of the two-step formation of nacre-like composite fibre. First step: a belt-shaped nacre-like fibre was obtained via a wet-spinning process. Second step: The polymer/nanoplatelets fibre was twist spun.

The fibres were obtained by a combination of drawing and twisting the fibre in its wet state (Fig. 1). This technique was similar to those previously reported for making threads from cotton^22^ as well as SWNTs^17^,^23^, graphene^13^ and Kevlar. Nevertheless, the fibres obtained in this study displayed essential morphological and functional differences compared with other fibres obtained from nanoscale components. The most important differences are that the platelet morphology of the inorganic components, rheological properties of the polymer–platelet mixture, and the addition of twisting during fibre processing imparts a multiscale organization to the fibres, leading to new deformation modalities and considerable improvement of mechanical performance.

Now we consider the fabrication process and different structural motifs in the fibres of the PVA/*G* composite. A stable, aqueous dispersion of *G* and PVA (Fig. 1, first step) was continuously extruded from a syringe, solidified by dehydration in an ethanol bath and drawn out using godet rollers. Similar to doctor blading, the sheer generated during twist drawing in the wet state results in self-organization of the nanosheets into the nacre-like brick-and-mortar architecture. The nanoscale layering was observed for all conditions tested, whereas the appearance of structural motifs at larger scale was depended on the content of organic phase in the composite. When the weight fraction of PVA was <30 wt% or >80 wt% (Supplementary Fig. 3a,h), the fibres showed helical ridges on their surfaces with a helical length periodicity of 220–300 μm (Supplementary Fig. 3a,h). When the PVA weight fraction was within this range, we observed additional coiling of the fibre, which resulted in a helical twist with a pitch of 100–200 μm (Supplementary Fig. 3b,d) and sub helical ridges of 0.92–20 μm (Supplementary Fig. 3e,g,i,j). Nearly perfect coiled fibres with all three structural motifs were obtained for PVA with weight fractions between 54 and 80 wt% (Supplementary Fig. 3c,f). The sub helical ridges on the surface were between 250 and 600 nm (Supplementary Fig. 3f).

Similar morphology of the fibres with nanoscale layering and two helical structured motifs can also be seen for PVA/CaCO_3_ composites (Supplementary Fig. 4). In this case, the sub helical ridges with a wavelength of about 6–12 μm were observed when the weight fraction of PVA was <50 wt% and >85 wt% (Supplementary Fig. 4a,e,c,g), while uniform 95–105 μm coils appear for PVA fractions between 50 and 85 wt% (Supplementary Fig. 4b,f). Furthermore, the wavelengths of the helical ridges on the coils were calculated to be in the range of 4.5–10 μm (Supplementary Fig. 4f).

### Morphology evolution during twist spinning

**Figure 2:**
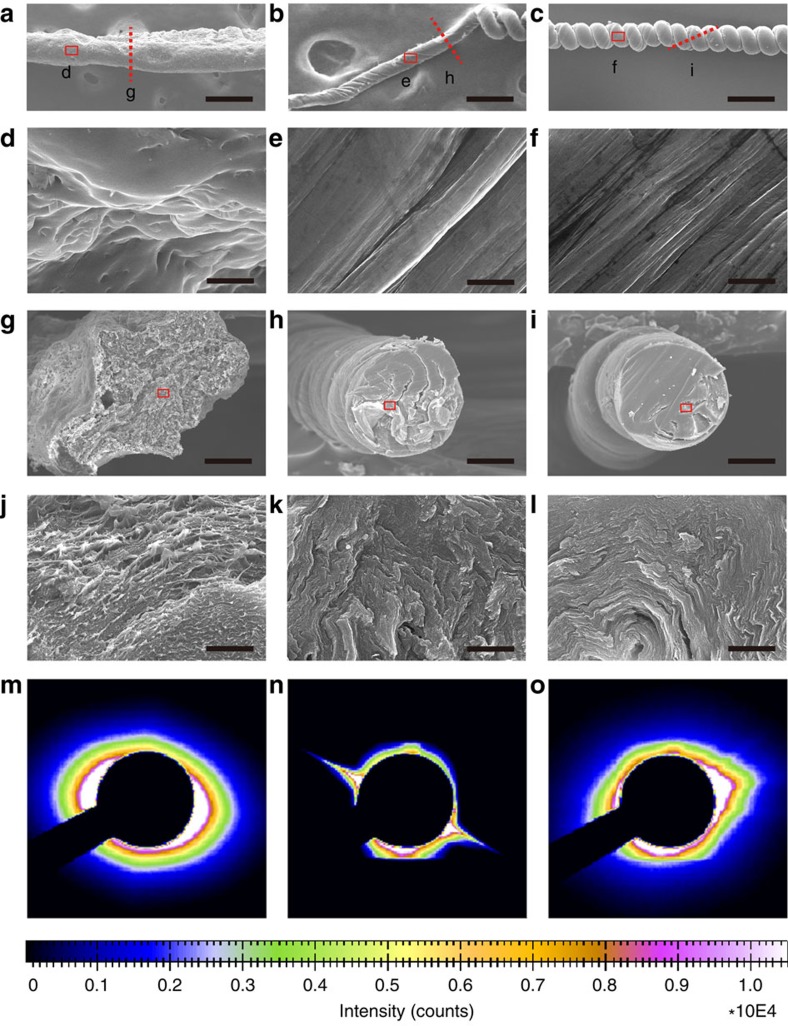
Characterizations of the microscale morphology evolution of PVA/*G* composite fibre with PVA content of 66 wt% during spinning process. Structural changes of PVA/*G* composite fibres during the production process: (**a**) belt-like fibre after drawing from the coagulation bath, (**b**) circular fibre after initial twist spinning, (**c**) spring-like fibres after additional twist spinning, (**g**–**i**) SEM images of cross section of the three types of fibre represented by dashed lines shown in **a**–**c**, (**j**–**l**) SEM images of cross–section marked in red boxes in **g**–**i**, (**m**–**o**) show S–SAXS scattering patterns of the PVA/*G* fibre with PVA content of 66%, for belt-like fibre (**m**) in **a**, twisted fibre (**n**) in **b** and coiled fibre (**o**) in **c**. All the specimens were vertically fixed. Scale bars in **a**,**b**,**c** are 250 μm, **d**,**e**,**f**,**j**,**k**,**l** are 2.5 μm, (**g**,**h**,**i**) are 50 μm, respectively.

To better understand the mechanical performance of the products, we investigated how the microscale morphology evolved after drawing them out of the coagulation bath. After extrusion of the PVA/*G* composite from a 400 μm diameter nozzle, a belt-like fibre (Fig. 2a), ∼225 μm in diameter, was formed; its nanoscale organization was characterized by disordered packing of the nanosheets (Fig. 2g). After twisting, the fibres became thinner (diameters ∼120 μm) and nearly circular in cross-section (Fig. 2b,h). Additional twisting further decreased the fibre diameters to ∼110 μm, and resulted in the formation of uniform, spring-like coils (Fig. 2c).

A similar progression of fibre shapes can be seen for the PVA/CaCO_3_ composite (Supplementary Fig. 5). Unlike the PVA/CaCO_3_ composite, the surface of belt-like fibres from *G* sheets had a non-uniform orientation of platelets (Fig. 2d). After the twist spinning, the fibres showed helical corrugations along the fibre axis with periodicity of 250–600 nm (Fig. 2e,f). The torque applied during spinning of the wet fibre resulted in considerable improvement of platelet alignment (Fig. 2j–l). Enhanced alignment of the platelets was also confirmed using synchrotron small-angle X-ray scattering (S–SAXS). 2D scattering patterns accompanied with the profile of scattering intensity (*I* × **q**^2^) as a function of scattering vector (**q**) (**q**=4*π*sin*θ*/*λ*) and profile of scattering intensity (*I*) as a function of azimuthal angle (*ϕ*) are given in Fig. 2m–o and Supplementary Fig. 6, respectively. Since PVA scatters X-rays weakly^24^, the diffraction peaks originated from the *G*. The elliptical S–SAXS pattern and absence of sharp scattering peak (Fig. 2m and Supplementary Fig. 6d) obtained for belt-like fibres before twisting indicated poor alignment of nanosheets^20^,^21^. The nearly perfect nacre-like layering in the transversal direction of the fibre indicated by the sharp scattering peak (Fig. 2n) was observed after twisting. This was accompanied by sharp peaks at 154^o^ and 329^o^, as shown in Supplementary Fig. 6d, which highlighted the strong alignment of platelets in the twisting fibre. Further twisting led to the formation of a coiled fibre and its longitudinal direction alignment (Supplementary Fig. 6c) resulted in an absence of a sharp peak in the 2D scattering pattern (Fig. 2o). The monotonous intensity drop with no visible peak (Supplementary Fig. 6e) is characteristic of the uniform dispersion of *G* in the PVA matrix for all of the samples^20^,^21^, which is both essential and non-trivial for composites with high content of *G*^25^,^26^,^27^. For both of the PVA/*G* and PVA/CaCO_3_ composites, the nanoscale structure of the fibres was analogous to the nacre-like materials previously reported^5^,^6^,^8^,^9^,^21^,^22^, with the exception of additional circular bending of the inorganic phase that becomes progressively more pronounced with increased coiling (Fig. 2j–l).

### Mechanical properties of nacre-like fibres

**Figure 3:**
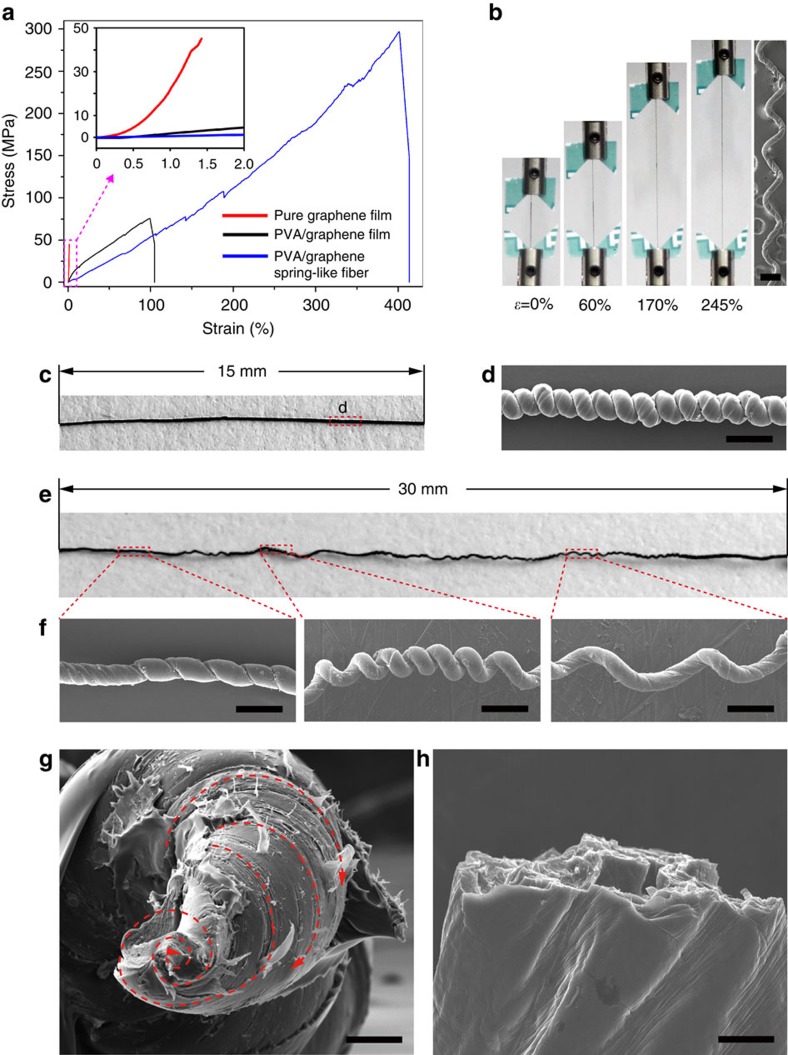
Mechanical properties and characterizations of spring-like PVA/G fibre with PVA content of 66 wt%. (**a**) A typical tensile stress–strain curve of neat *G* film, PVA/*G* film, and spring–like fibre, (**b**) Photography of the fibre at strains of 0%, 60%, 170% and 245%, respectively. The right panel shows the SEM image of extended loops after breaking, bar, 250 μm, (**c**,**e**) Photographs of a 15-mm-long spring-like fibre before and after being stretched to 200%, showing a partial opening of loops, (**d**,**f**) Enlarged SEM images taken from different parts of the fibre, (**g**,**h**) Typical tensile fracture surface SEM image of spring-like fibre and belt-like fibre, the dash arrows in **g** show the progressive cone–shaped surface. Scale bars in **d** and **f** are 250 μm, **g** and **h** are 50 μm, repsectively.

Mechanical properties of the fibres were assessed using the stress–strain test (Fig. 3a). The tensile strength was 270±30 MPa, which was comparable to other nacre-like materials made from clay, graphene or graphene oxide (GO). Herein, the tensile stress was calculated from the normalization of the applied force with a cross–section of microfibre after fracture. More importantly, elongation to fracture, that is, maximum tensile strain (*ɛ*), was as high as 330±60%; this compares favourably to tensile strains of natural nacre (*ɛ*=2%)^9^, dragline silk (*ɛ*=31–47%)^14^, graphene composites (*ɛ*=1.6–76%)^13^,^28^, Kevlar(KM2) (*ɛ*=4.15–4.89%)^14^ and SWNT composite fibres (*ɛ*=104–430%)^15^,^16^. The area under the stress-strain curve was used to calculate toughness, which was determined to be 460.3±42 MJ m^−3^ and 168.1±18.2 MJ m^−3^ for *G* (Fig. 3a) and vaterite fibres (Supplementary Fig. 7), respectively. In contrast, for an equivalent PVA fraction of 66 wt%, belt-like and circular fibres dissipated only 46.3±5.4 MJ m^−3^ and 118.2±9.7 MJ m^−3^, respectively, before fracture. Because the density of our fibres is ∼0.84 g cm^−3^, the weight-specific toughness of the material was calculated to be 548.0±50 J g^−1^. For comparison, tensile strength, *ɛ* and toughness for ‘flat' graphene paper obtained by vacuum assisted filtration (VAF)^29^ without polymer were reported to be 41.7±4 MPa, 1.34±0.07% and 0.23±0.03 J g^−1^, respectively; the same values for PVA/graphene nanocomposites prepared via layer-by-layer assembly^29^ were 143.1±11.2 MPa, 0.051±0.013% and 6.1 MJ m^−3^, respectively. We also prepared a PVA/*G* composite using the VAF method with 66 wt% PVA (Supplementary Fig. 8). It showed tensile strength, *ɛ* and toughness of 65.8±12 MPa, 95.5±8% and 48.6±6 J g^−1^, respectively. The maximum strain sustained by the nacre-like PVA/*G* fibre is ∼246 times higher than that of the neat *G* film or approximately 3.5–4 times higher than that of nacre-like flat PVA/*G* composite film (VAF) with the same *G* loading. The toughness of spring-like PVA/*G* fibre is ∼2,380 and 11 times higher than that of the *G* paper and ‘flat' 66% PVA/*G* composite film made by VAF, respectively. Similarly, outstanding mechanical properties were also obtained for PVA/CaCO_3_ fibres (Supplementary Fig. 7a), which showed a strain of 200.6±14%, and toughness of 107.1±11.6 J g^−1^, with a fibre density of ∼1.57 g cm^−3^.

**Figure 4:**
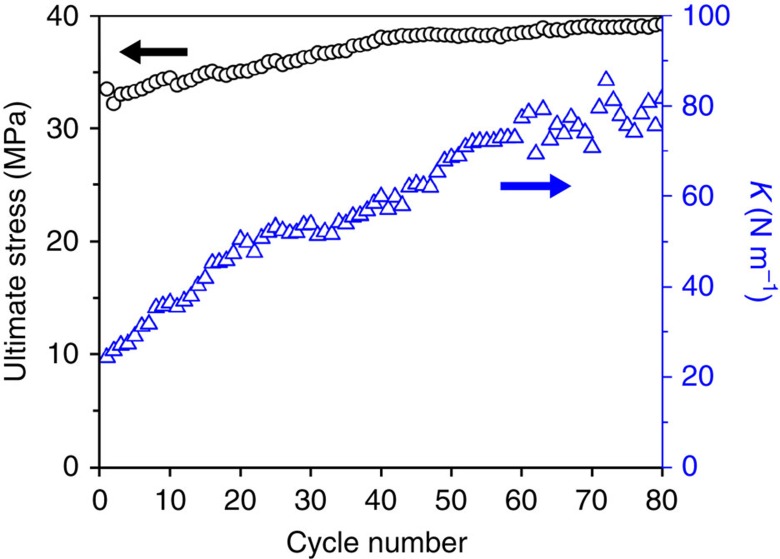
Tensile testing. Tensile stress and spring constant (*k*) of PVA/G fibre at *ɛ*=20% as a function of the cycle number during cyclic tensile testing.

A cyclic stretching test with *ɛ*=20% was used to investigate the strain-hardening processes of the nacre-like fibres (Supplementary Fig. 9a). For the first to 50th cycles, the PVA/*G* fibres exhibited considerable plastic deformation typical of many biological and synthetic materials^30^,^31^. After the 50th cycle, the deformation became mostly elastic (Fig. 4). The tensile stress for ɛ=20% saturates at ∼39 MPa for the 80th cycle (Fig. 4 and Supplementary Fig. 9b), which is about 1.18 times its initial value. Strain hardening was accompanied by tighter packing of the nanosheets and an increase of their curvature (Supplementary Fig. 9c–f). After being pre-stretched, the fibre still showed the visible hysteresis between the loading and unloading curve with energy dissipation of 0.13 J g^−1^ (Supplementary Fig. 10), indicating viscoelastic behaviour of the nacre-like fibre. Notably, the spring constant (Supplementary Fig. 11) after strain hardening was 76.8±1.8 N m^−1^, twice as much as conventional steel-based tension springs and carbon nanocoils^32^.

It is informative to compare the mechanical properties and toughness of the obtained nacre-like fibres with other well-known materials, such as dragline silk (165 J g^−1^)^14^ and Kevlar(KM2) (78 J g^−1^)^14^, as well as additional human-made fibres exemplified by various carbon nanotube- and graphene-based composites (Supplementary Table 1). Note that while nacre is known for its toughness of ∼1 J g^−1^, the twisted PVA/CaCO_3_ composite fibres are over ∼107 times tougher.

It is also instructive to compare the mechanical properties of the prepared twisted fibres with those made from neat PVA using the same apparatus. The maximum strain and toughness of neat PVA fibre without *G* are 102.6±14% and 13.6±3.8 J g^−1^, respectively. This fact is remarkable because parent polymeric materials typically exhibit higher stretchability than their resulting composites. Both vaterite and *G* composites display greater strains than their parent polymers. The fibre's mechanical properties for different mass fractions of PVA peak at about 65% PVA (Supplementary Fig. 12).

**Figure 5:**
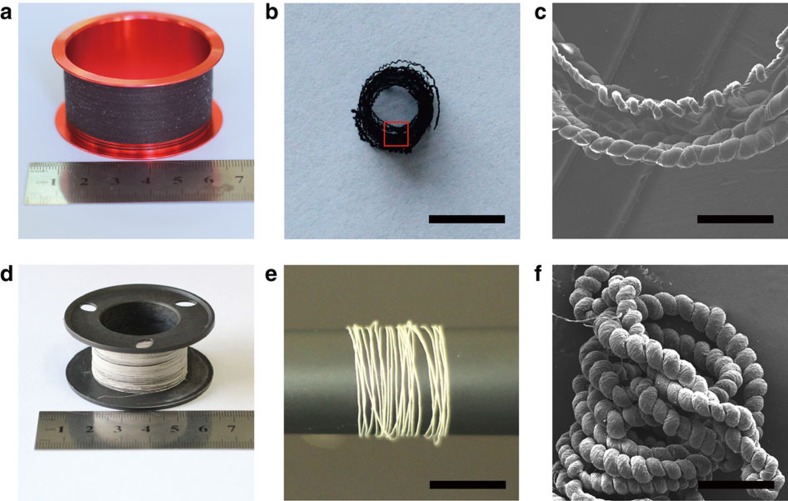
Characterization of a coil of PVA/*G* and PVA/CaCO_3_ fibre. (**a**) Photograph of PVA/*G* fibre wound on a reel, (**b**) Top view: a coil of spring-like PVA/*G* fibre, (**c**) SEM image of coiled spring-like PVA/*G* fibre marked in box in **b**, (**d**) Photograph of PVA/CaCO_3_ fibre wound on a reel, (**e**) A coil of spring-like PVA/CaCO_3_ on a roll, (**f**) SEM image of a coil of PVA/CaCO_3_ fibre. Scale bars in **b** and **e** are 4 mm, **c** and **f** are 0.5 mm, respectively.

The fibres can be made by a continuous mode, forming rolls of nacre-like fibrous material (Fig. 5) with identical morphology over the entire fibre, exemplified by a 4 cm long fibre, displaying uniform diameter and pitch over its entire length (Supplementary Fig. 11b).

### Circularly polarized emission of helical fibres

**Figure 6:**
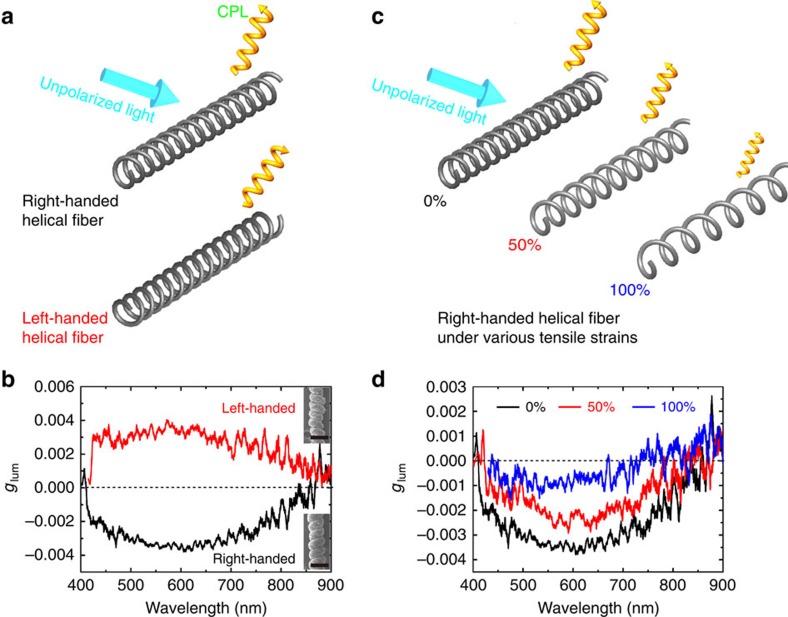
Circularly polarized luminescence characteristics of PVA/CaCO_3_/CdTe helical fibres. (**a**) Schematic illustration of different circularly polarized luminescence (CPL) emissions of left- and right-handed helical fibres under illumination of unpolarized light, (**b**) g_lum_ spectra of left- and right-handed helical fibres. Inset SEM images show a representative left-handed helical fibre (top) and a right-handed helical fibre (bottom), scale bar, 200 μm, (**c**) Schematic illustration of CPL activity from luminescent helical fibres at different tensile strains of 0, 50 and 100%, (**d**) *g*_lum_ spectra of right-handed helical fibres at various stretching states (*ɛ*=0, 50, 100%).

Luminescent helical fibres were prepared by incorporating CdTe nanowires into PVA/CaCO_3_ composites during the spinning process (Methods section). The content of CdTe nanowires was about 0.9 wt%, as measured by thermogravimetric analysis (Supplementary Fig. 15), and the fibres retained their original morphology and stretchability. Both left- and right-handed helical fibres can be produced through controlling the spinning direction (Fig. 6b inset). Strong CPL of these fibres was observed under the illumination of non-polarized light. Shifting the excitation wavelength by 20 nm (from 358 to 338 nm) did not change the peak maxima of the corresponding photoluminescence spectra (Supplementary Fig. 16), which demonstrated that Raman scattering was not a major contributor to these chiroptical properties and confirmed the photoluminescence origin of the observed spectra when the fibres are irradiated with CPL light. Consecutive measurements of the same fibres yielded identical *g*_lum_ spectra (Supplementary Fig. 17), confirming that these fibres are relatively optically robust when irradiated.

The luminescence dissymmetry ratio (*g*_*l*um_) was calculated as


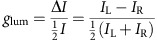


where *I*_L_ and *I*_R_ represent left- and right-handed CPL, respectively. Fibres of opposite handedness (Fig. 6a) exhibit almost mirror-imaged *g*_lum_ spectra along the *x* axis with peak maxima of similar intensities: 3.7 × 10^−3^ (580 nm) for the right-handed fibres and 4.0 × 10^−3^ (572 nm) for the left-handed fibres (Fig. 6b). The value of *g*_lum_ is comparable to that observed for other semiconductor materials such as cysteine-capped CdSe nanoparticles^33^ (3 × 10^−3^ and 4 × 10^−3^) and CdS nanoparticles templated in a protein nanocage^34^ (4.4 × 10^−3^), though our macroscale twisting process is considerably simpler.

CPL activity is associated with the helicity of the fibres. An effective validation of this mechanism would be modulation of CPL activity by fibre helicity changes induced by a mechanical deformation operation such as stretching. Indeed, the *g*_lum_ spectra of luminescent spring fibres show a strong dependence of CPL attenuation on the fibre elongation (Fig. 6c,d and Supplementary Fig. 18). As the fibres are stretched from their original length to 1.5 and 2 times their original length, the *g*_lum_ at peak maxima is decreased from 3.7 × 10^−3^ to 2.8 × 10^−3^ and finally to 1.3 × 10^−3^ (Fig. 6c,d). This mechanical manipulation of CPL emission is efficient in a broad illumination wavelength range of 400–900 nm (Fig. 6d), showing that smaller *g*_lum_ values can be obtained from the increasingly stretched fibres.

## Discussion

Previous versions of coiled fibres obtained by spinning and scrolling carbon nanotubes and GO films dissipated energy predominantly via macroscale deformation of the coils and displayed toughness of 28.7 J g^−1^ and 17 J m^−3^, respectively^13^,^35^. Other deformation modalities involving, for instance, nanoscale deformations, might be expected but were not investigated. This may be partially related to less pronounced structural changes than those observed in Figs 2 and 3, and the related inability to use SAXS to visualize the structural evolution under strain.

The unusually high toughness for nacre-like fibres in Figs 1 and 2 is attributed to multiscale deformation of the fibres on strain that involves both sliding/reorganization of the nanoscale sheets and microscale deformation of the coils. Indeed, opening of the coils and decreasing coil density can be seen as the strain increases from 0 to 60%, 170% and 245% (Fig. 3b). Various degrees of coil opening along the length of the fibre (Fig. 3c–f) suggests somewhat unequal strain along the fibre during stretching.

The combination of nanoscale and microscale deformations allows the material to experience strong intermolecular interactions between the nanosheets and the polymers, while uniformly distributing the stress along the length of the fibre. While the former mechanism is possible for ‘flat' nacre-like composites, the latter, or uniform stress distribution, is not. Minor defects result in stress concentration in the small volumes of the material and consequent failure. Other processes to achieve high elongation and thereby toughness include using elastic polymers or composites^17^,^36^, taking advantage of the intrinsic elasticity of polymers^36^ or interconnected networks and chemical bonds of constituents^17^. While yielding high-performance materials, the mechanism of energy dissipation in these fibres is different from that described for fibres with *G*, CaCO_3_ and CdTe.

Distribution of stress over the extended volume of the material in helical fibres can be seen from the conical shape of the fracture surface (Fig. 3g). The cracks apparently do not propagate perpendicularly to the surface but do so parallel to the length of the fibre, which dissipates more energy. In contrast, the fracture surface in the belt-like fibre (Fig. 3h) is perpendicular to the fibre axis. Consequently, tensile strength, strechability and toughness of the helical fibres are about 4, 4 and 11.5 times higher than those of belt-like fibres, respectively (Fig. 3a). Similar tensile deformation behaviour can also be observed in the PVA/CaCO_3_ composite fibre (Supplementary Fig. 7b,c).

The same multiscale deformations result in a novel chiroptical functionality: CPL. Circular asymmetry of emission is possible but difficult to obtain for optically active compounds including quantum dots^33^, nanofibres^37^, liquid crystals^38^ and molecular luminophores^39^. In our case, CPL is not only strong in intensity and simple to realize, but it can also be effectively modulated by the macroscopic structures of the fibres including handedness and pitch. When the handedness of the host fibres was changed from left-handed to right-handed, there was a corresponding sign inversion of CPL from positive to negative (Fig. 6). The mechanism of *g*_lum_ modulation by deformations can be associated with strain-dependent variation of pitch of the fibre resulting in the transfer of chirality to smaller scales affecting the three-dimensional geometry of the nanowires in the matrix and efficiency of emission of left and right circularly polarized photons. Mechanical manipulation of CPL can be compared with other methods of its modulation including phase transition of liquid crystals^38^, varying aggregation states^37^,^40^, temperature^38^, chemical stimuli^41^ and solvent^42^.

We demonstrated that multiscale deformations in nacre-like fibres have important ramifications for both mechanical and optical properties of the nacre-like fibres. One of them is exceptional toughness while the other one is emergence of optical asymmetry for circularly polarized photons. Since strain-induced particle self-organization can be realized for a variety of nanoscale components^43^,^44^, the method of fibre preparation can be generalized to other inorganic nanomaterials for mechanically tunable CPL-active composites and enables a novel technologically significant method to manipulate the chiroptical activity of materials that can be utilized among other applications for the stand-off assessment of mechanical strains.

## Methods

### Electrochemical exfoliation of graphene-based nanosheets

Highly oriented pyrolytic graphite (HOPG, 1 × 1 × 0.3 cm) was employed as the electrode and source of graphene for electrochemical exfoliation. The anode was a block of HOPG, gripped and inserted into the electrolyte. A Pt plate served as the grounded electrode, and was placed parallel to the HOPG with a separation of about 4 cm. The electrolyte was prepared by taking 2.6 ml fuming sulfuric acid and diluting in 100 ml deionized water and then a 30% potassium hydroxide solution was added drop by drop to neutralize the sulfuric acid until the pH=∼1.2. The electrochemical exfoliation process was carried out by applying DC bias on the HOPG electrode (from −10 V to +10 V). When the HOPG was completely exfoliated, *G* nanosheets were collected by vacuum filtration and rinsed at least three times with deionized water. After drying, the *G* was re-dispersed in dimethyl formamide (DMF) solution by gentle sonication. Finally, the suspension was centrifuged at 2,500 r.p.m. for 3 min to remove blocked graphite. The centrifuged suspension was later used for further characterization.

### Characterization of low defect graphene–based nanosheets

Supplementary Figure 2a shows an scanning electron microscopy (SEM) image of *G* made by electrochemical exfoliation of HOPG on a SiO_2_/Si substrate. The sheet sizes range from 5 to 45 μm. Supplementary Figure 2b shows a typical atomic force microscopy (AFM) image of *G* sheet on a SiO_2_/Si substrate with a thickness of ∼2.8 nm (Supplementary Fig. 2c). We have measured tens of *G*s and their thicknesses ranged from 1 to 5 nm, indicating that the number of layers in *G* is less than five.

*G*s show smaller density of defects than conventionally used GO prepared by modified Hummers' method^19^. We have compared the Raman spectra of *G* sample with that of the original graphite and GO sample^19^ (Supplementary Fig. 2d). The Raman spectrum of the *G* sample is very different from that of a GO sample. The appearance of D band indicates that there are still defects (disorder sp^3^ structure, oxygen-contained functional groups) in *G* plane or at edges, which may originate from some oxidation of diluted sulfuric acid and positive electrical potential. The intensity ratio of D band over G band (*I*_D_/*I*_G_) was calculated to be 0.08, for the *G* is incomparable to negligible defect signal in graphite but much smaller than that of GO sample (*I*_D_/*I*_G_=1.12). According to Tuinstra and Koenig^45^, *I*_D_/*I*_G_ varies inversely with the crystallite size (*L*_a_) in graphite: *L*_a_ (nm)=2.4 × 10^−10^ × *λ*^4^(*I*_D_/*I*_G_)^−1^, where *λ* is the Raman excitation wavelength. Using this relationship, the crystallite sizes (graphitic domain size) of GO and as-prepared *G* are 18.3±2.0 and 97.4±43.8 nm, respectively. Moreover, the *I*_2D_/*I*_G_ has been proved to be related to the degree of sp^2^ structure in graphite, herein, the ratio of 2D band over G band (*I*_2D_/*I*_G_) for the graphene sample is significantly larger than that of GO sample. Furthermore, we have measured the thermogravimetric curves of the *G*, GO, pure PVA and PVA/*G* samples (Supplementary Fig. 2e). The GO sample shows a weak thermal stability, the mass loss starts once heating begins and is dramatic until 250 °C, which is similar to the results reported by Stankovich *et al*^46^. The mass loss in GO sample is attributed to pyrolysis of oxygen groups such as –OH, –O–, –COOH and so on, the higher defect density present in GO, the lower starting temperature for rapid mass loss and *vice versa*. Otherwise, a decrease in defect density would improve the thermal stability of *G*. The weight decreases slowly while the temperature is above 250 °C and approaches to 40% at 800 °C. But for the *G*, there is continuous mass loss in the whole range of heating and keeps ∼91% mass at 800 °C. Notably, *G* possesses higher thermal stability than the GO indicating a lower defect in the *G* sheet, also. The small changes in weight of the *G* can be attributed to the release of absorbed water in the surface of the nanosheets.

### Synthesis of CaCO_3_ nanoplatelets

The CaCO_3_ nanoplatelets were prepared as follows: 2 g of CaCl_2_ and 2 g of urea were added into 6.0 ml of deionized water and stirred vigorously. Then 0.44 g of hexadecyl–trimenthyl–ammonium bromide was added into the mixture, followed by adding 50 ml of ethylene glycol and 200 μl of Tween 20. The entire solution was transferred to a Teflon-lined stainless steel autoclave, and then was stirred for an additional 30 min. Finally, the autoclave was placed inside an electric oven at 140 °C for 4 h. After the reaction, the autoclave was cooled, and the suspension was centrifuged at 6,000 r.p.m. for 5 min. The centrifugation and redispersion cycles were repeated twice with deionized water and ethanol, respectively. The precipitate was oven dried at 60 °C for 12 h.

Supplementary Figure 1a shows the typical SEM image of as-synthesized CaCO_3_ nanoplatelets, the lateral size is in the range of 4–20 μm with several to tens of layers. X-ray diffraction pattern (Supplementary Fig. 1b) shows that the products are mainly vaterite with a trace amount of aragonite and calcite (JCPDS 721652, lattice constants *a*_0_=4.99 Å and *c*_0_=17.00 Å, space group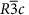)^47^. The thickness of a single CaCO_3_ nanosheet is in the range of 100–500 nm with the average of ∼320 nm (Supplementary Fig. 1c and d), which is in the same scale as that for natural nacre^4^,^5^,^9^.

### Synthesis of TGA–CdTe NPs

Cd(ClO_4_)_2_·6H_2_O (0.985 g) and thioglycolic acid (TGA, 0.392 ml) were dissolved in 125 ml of deionized water, followed by adjusting the pH to 11.2 with 1 M NaOH. This solution was placed in a three-neck round-bottom flask and purged with N_2_ for 30 min. H_2_Te gas (generated by reacting 0.13 g Al_2_Te_3_ with 25 ml of 0.5M H_2_SO_4_) was slowly passed through the solution. The solution was then allowed to reflux under N_2_ at 100 °C for 120 min to obtain the TGA–CdTe NPs (Supplementary Fig. 13).

### Preparation of TGA–CdTe nanowires

15 ml of TGA–CdTe NP solution was mixed with 22.5 ml of methanol, yielding red precipitation in the solution. After centrifuging at 1,500 r.p.m. for 3 min, the supernatant was removed and the precipitation was re-dispersed in deionized water and pH adjusted to 9 by the addition of 0.1 M HCl solution. The solution was aged in the dark at room temperature and nanowires (Supplementary Fig. 14) were formed after 6 days of aging.

### Preparation of nacre-like PVA/*G* composite fibres

For preparation of PVA/*G* composite fibres, dried and powdered *G* was re-dispersed in deionized water by gentle sonication; the concentration was 10 mg ml^−1^. PVA aqueous solution with a mass fraction of 5 wt% was prepared from dissolved PVA (molecular weight= ∼205,000, hydrolysis ≤89%, Aladin) in deionized water. For preparing 66 wt% PVA/*G* mixture, 2.7 ml 5 wt% PVA aqueous solution was added to 7.3 ml *G* suspension (10 mg ml^−1^) and stirred at 600 r.p.m. for 12 h. Then the mixture was concentrated by heating until a final mixture volume of 7 ml. Other ratios of PVA in the mixture was regulated by adding different volumes of 5 wt% aqueous PVA. For the wet-spinning process, first the concentrate was loaded into a 50 ml plastic syringe with a spinning nozzle (diameters=100, 200 and 400 μm), and injected into a container with 300 ml coagulation bath (for example, ethanol with 5 wt% NaOH, acetone, Na_2_SO_4_) by a syringe pump at speed of 0.05–3 ml min^−1^. After coagulation for 0.5–5 min, the fibres were drawn out and transported by godet roller 1. For the second step, the PVA/*G* fibre was fixed and fed into the central axis of the twister by godet rollers 1 and 2. Then, the fibre was twisted into a spring-like shape and collected by the drum. Godet roller 2 was fixed on the twister and rotated with it, which kept the fibre in the central axis of the twister throughout the process. Usually, as-spinning fibre began to transfer to coiling fibre after twisting 33–36 circles per cm, then another 70–85 circles per cm twisting was needed to form a complete loop. Finally, the average density of loops was in the range of 10–25 loops per mm. We have prepared a series of mass fraction of PVA/*G* solutions (for example, 0, 4.4, 11 30, 54, 66, 80, 90 and 100 wt%) for spinning fibres. For comparison, *G* composite films with various PVA content were prepared by VAF.

### Preparation of PVA/CaCO_3_ composite fibres

The spinning process is the same as that of PVA/*G* detailed in Fig. 1. In brief, 0.4 g dried CaCO_3_ nanoplatelets were added in 3.5 ml of ethanol and 5.0 ml of deionized water and stirred for 20 min. Then, 8 g of 5 wt% of aqueous solutions of PVA was added into the CaCO_3_ solution and stirred for 24 h. The solution was centrifuged at 3,500 r.p.m. for 3 min. The sediment was re-dispersed in a little deionized water, and stirred vigorously (the content of CaCO_3_ was about 0.2–0.4 g ml^−1^). Then the mixture was injected into 5 wt% of ethanol solution of sodium hydroxide (the total volume was 200 ml) and a fibre was formed. To curdle the fibre, it was kept for 15 min in a coagulation bath. The wet fibre was dried at 60 °C for 1 h under vacuum. Twist-spinning process was similar to that for the PVA/*G* spring fibre.

### Preparation of PVA/CaCO_3_/CdTe composite fibres

The spinning process is the same as that of PVA/CaCO_3_ fibre detailed in Fig. 1. In brief, 0.4 g dried CaCO_3_ nanoplatelets were added in 3.5 ml of ethanol and 5.0 ml of deionized water and stirred for 20 min. Then, 8 g of 5 wt% of aqueous solutions of PVA and 5 ml as-prepared CdTe nanowire solution was added into the above solution and stirred for 24 h. The solution was centrifuged at 3,500 r.p.m. for 3 min. The sediment was re-dispersed in a little deionized water, and stirred vigorously (the content of CdTe is about 0.9 wt% thermogravimetric analysis curve in Supplementary Fig. 15). Then the mixture was injected into pure ethanol solution and a fibre was formed. To curdle the fibre, it was kept for 15 min in a coagulation bath. Twist-spinning process was similar to that in the PVA/*G* spring fibre.

### Characterization

SEM images were obtained using a Hitachi SU–8000 with accelerating voltage of 15 kV. AFM images were acquired in the tapping mode with a commercial multimode Nanoscope IIIa (Veeco Co., Ltd.). Raman spectra were collected using a LabRAM XploRA laser Raman spectroscope (HORIBA Jobin Yvon Co., Ltd.) using a 532-nm laser with incident power of ∼1 mW. The *G* samples used in SEM, AFM and Raman characterizations were transferred to a silicon substrate that had a 300 nm thermal oxide layer. Thermogravimetric analysis was conducted in a nitrogen atmosphere from room temperature to 800 °C with a heating rate of 20 °C min^−1^. About 5 mg of each sample was used for the tests. SAXS analyses were carried out at the Shanghai Synchrotron Radiation Facility, using a fixed wavelength of 1.24 Å, a sample-to-detector distance of 5,180 mm and an exposure time of 10 s. The scattering patterns were collected on a CCD camera. The area of the incident X-ray spot was 400 × 600 μm^2^. Mechanical tests were carried out using the tensile tester equipped with a single column (Agilent Technologies T150UTM). For the test, each end of the fibre was first fixed on a paper with a cut window and then the paper was cut from two sides to free the fibre. For tension tests, the upper grip moved at a constant strain rate of 2.7 × 10^−3^ s^−1^. All of the mechanical tests were carried out at a relative humidity of 50% and room temperature. All of the data were reported with a 95% confidence level. CPL studies: JASCO CPL–300 was used for CPL measurements. Emission intensity (arbitrary units), represented by the direct current voltage collected by the instrument, was measured along with CPL (unit: mdeg). Typical CPL settings were as follows: excitation wavelength=358 nm, data pitch=0.1 nm, scanning speed=50 nm min^−1^, digital integration time=8 s and 10 accumulations. The luminescence dissymmetry ratio *g*_lum_ was calculated using the following equation: *g*_lum_=0.00006978 × CPL/emission. As-prepared spring fibres (coagulated in ethanol) incorporating CdTe nanowires were used for CPL studies. Multiple strands of the original spring fibres were tethered onto the sample holder so that they aligned vertically along the centre of the irradiation beam. The original fibres were slowly stretched after a 30-s immersion in a mixed solvent system of water and ethanol with a volume ratio of 1:9, followed by re-attachment to the sample holder for subsequent CPL measurements.

## Additional information

**How to cite this article:** Zhang, J. *et al*. Multiscale deformations lead to high toughness and circularly polarized emission in helical nacre-like fibres. *Nat. Commun.* 7:10701 doi: 10.1038/ncomms10701 (2016).

## Supplementary Material

Supplementary Information Supplementary Figures 1-18, Supplementary Table 1 and Supplementary References
