# Supplementary material for: Multiscale deformations lead to high toughness and circularly polarized emission in helical nacre-like fibres
Source: Nat Commun. 2016 Feb 24;7:10701. doi: 10.1038/ncomms10701 (PMC4770083; doi:10.1038/ncomms10701)
Supplement: Supplementary Information — Supplementary Figures 1-18, Supplementary Table 1 and Supplementary References [file ncomms10701-s1.pdf]

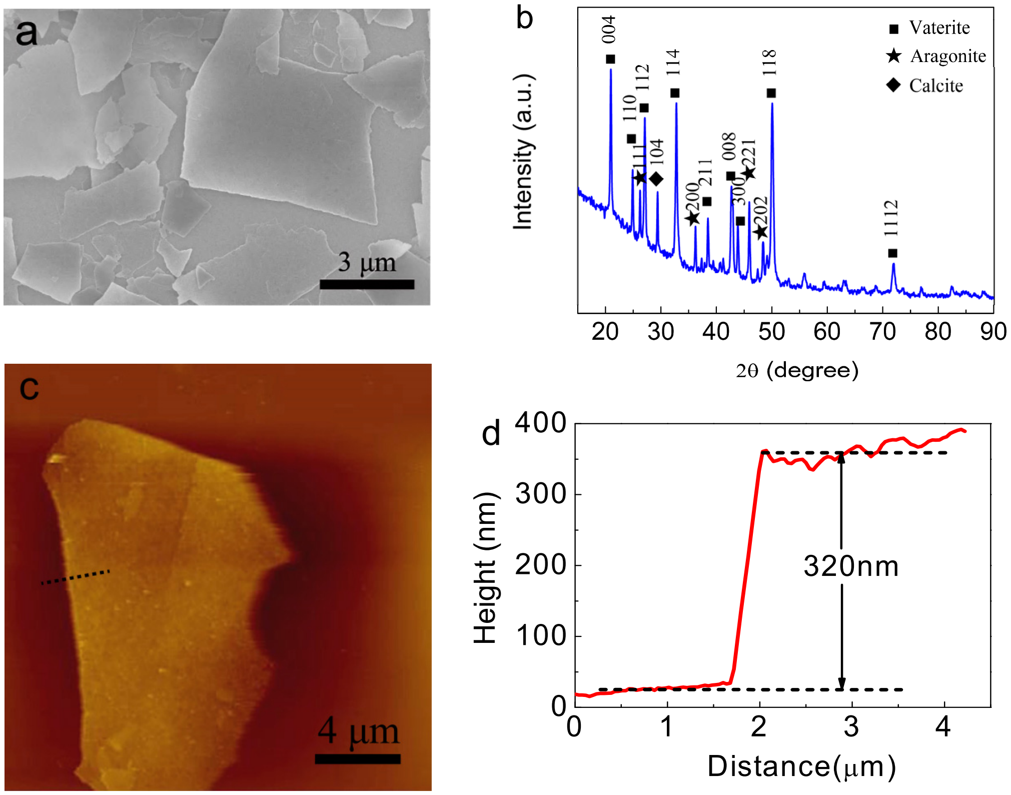

Supplementary Figure 1 Characterization of as–synthesized $CaCO_3$ nanoplatelets. (**a**) SEM image, (**b**) XRD pattern, (**c**) Typical AFM image of a single $CaCO_3$ nanoplatelet, (**d**) Height profile along the dashed line in Supplementary Fig. 1c and the thickness of $CaCO_3$ nanoplatelets is ~320 nm.

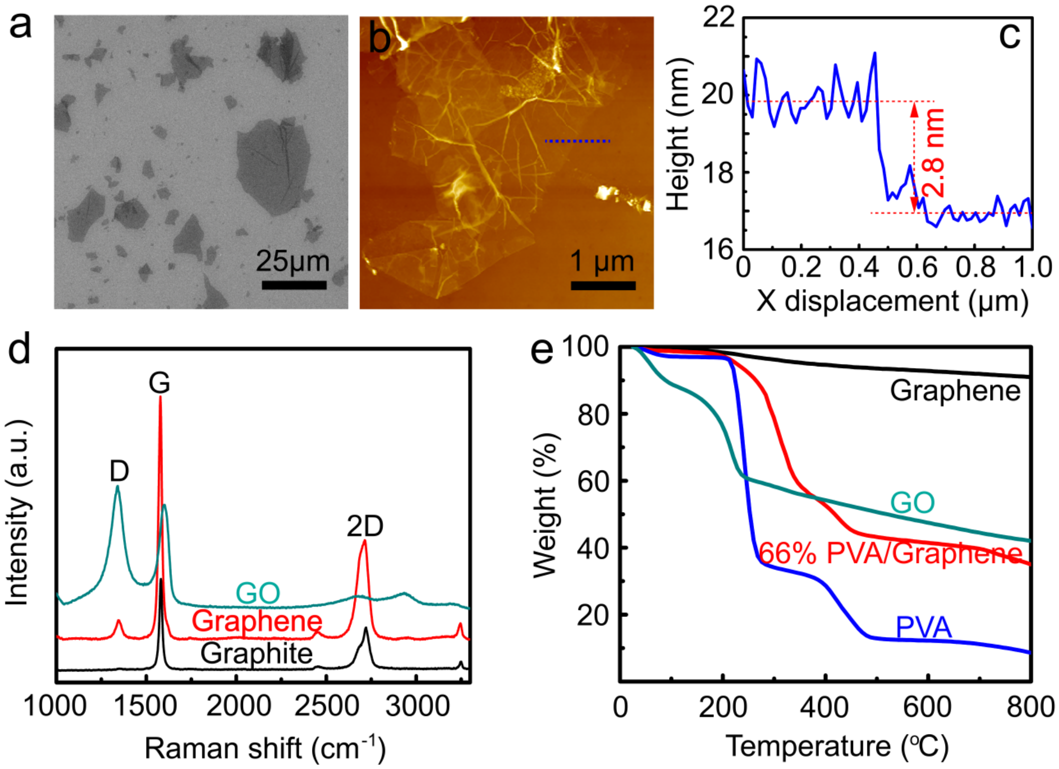

Supplementary Figure 2 Characteriation of as-synthesized graphene sheets (*G*). (**a**) SEM image of *G* spin–coating on a SiO_2/Si substrate, the lateral size of *G* is in the range of 5–45 µm, (**b**) Typical AFM image of *G* on SiO_2/Si substrate, (**c**) Height profile along the line in Supplementary Fig. 2b and the thickness of *G* is ~2.8 nm, (**d**) Raman spectra of *G*, GO, and original graphite samples, (**e**) TGA curves of the *G*, GO, pure PVA, and 66 wt% PVA/*G* composite samples.

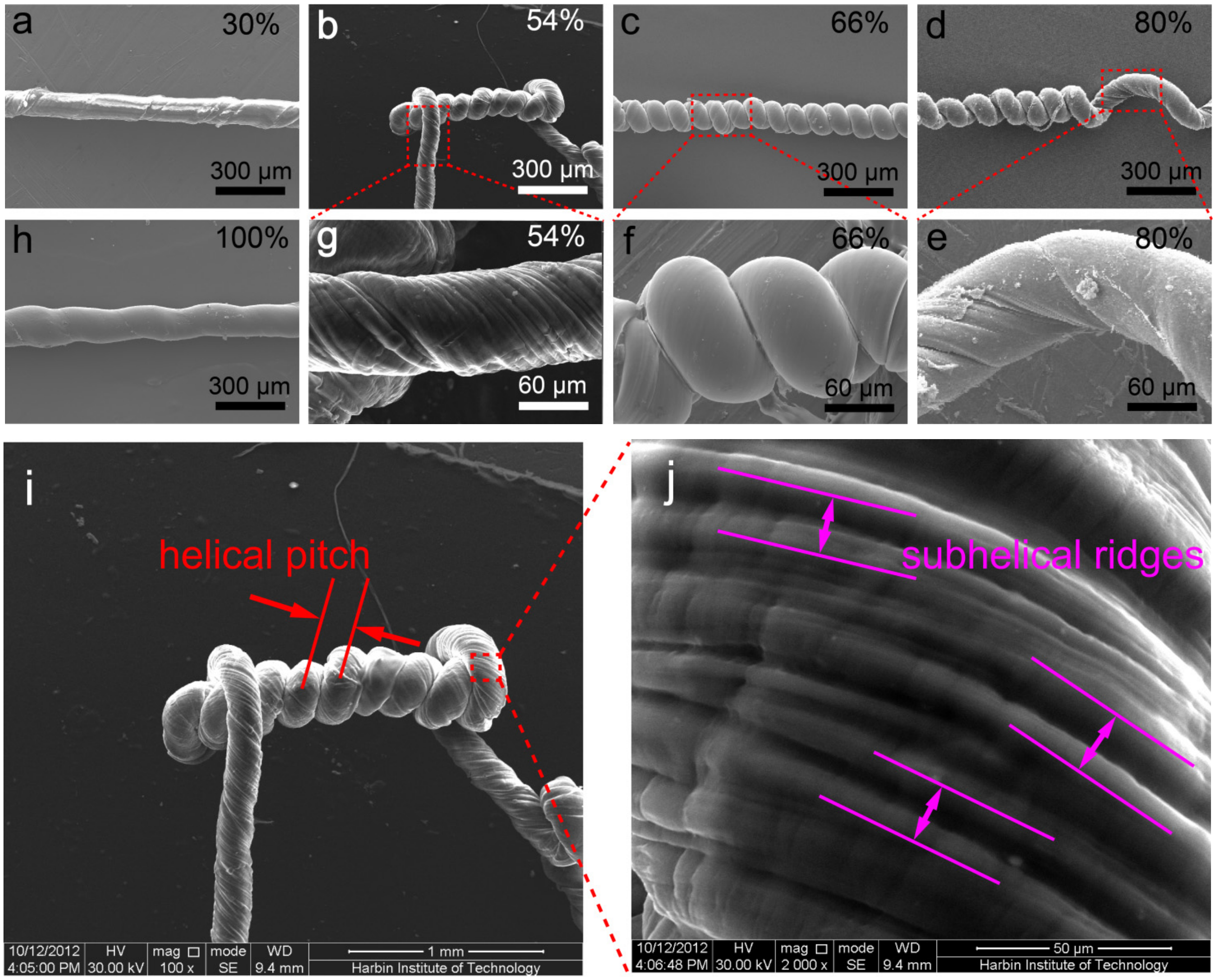

Supplementary Figure 3 SEM image of PVA/*G* fiber with different PVA weight fractions of (**a**) 30% and (**b**, **g**) 54%, (**c**, **f**) 66%, (**d**, **e**) 80%, and (**h**) 100%. Enlarged SEM image of (**e**, **f**, **g**) show the surface helical ridges in the fibers. Illustration of the helical pitch (**i**) and sub helical ridges (**j**) of our coiled fiber.

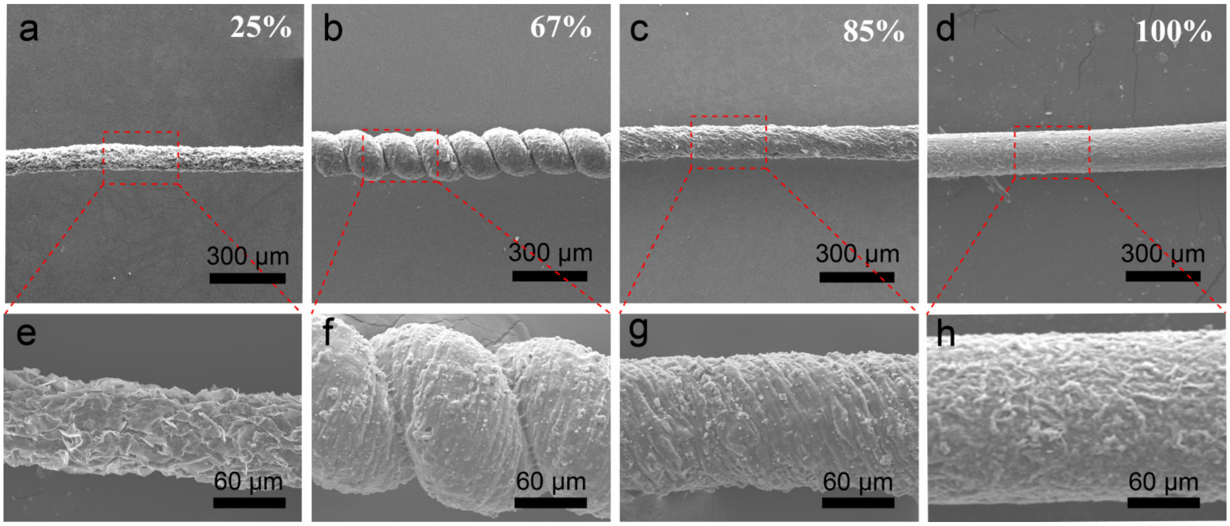

Supplementary Figure 4 SEM image of PVA/$CaCO_3$ fiber with different PVA weight fractions of (**a**) 25% and (**b**) 67%, (**c**) 85%, and (**d**) 100%. Enlarged surface SEM image of fiber with PVA weight fractions of (**e**) 25%, (**f**) 67%, (**g**) 85%, and (**h**) 100%.

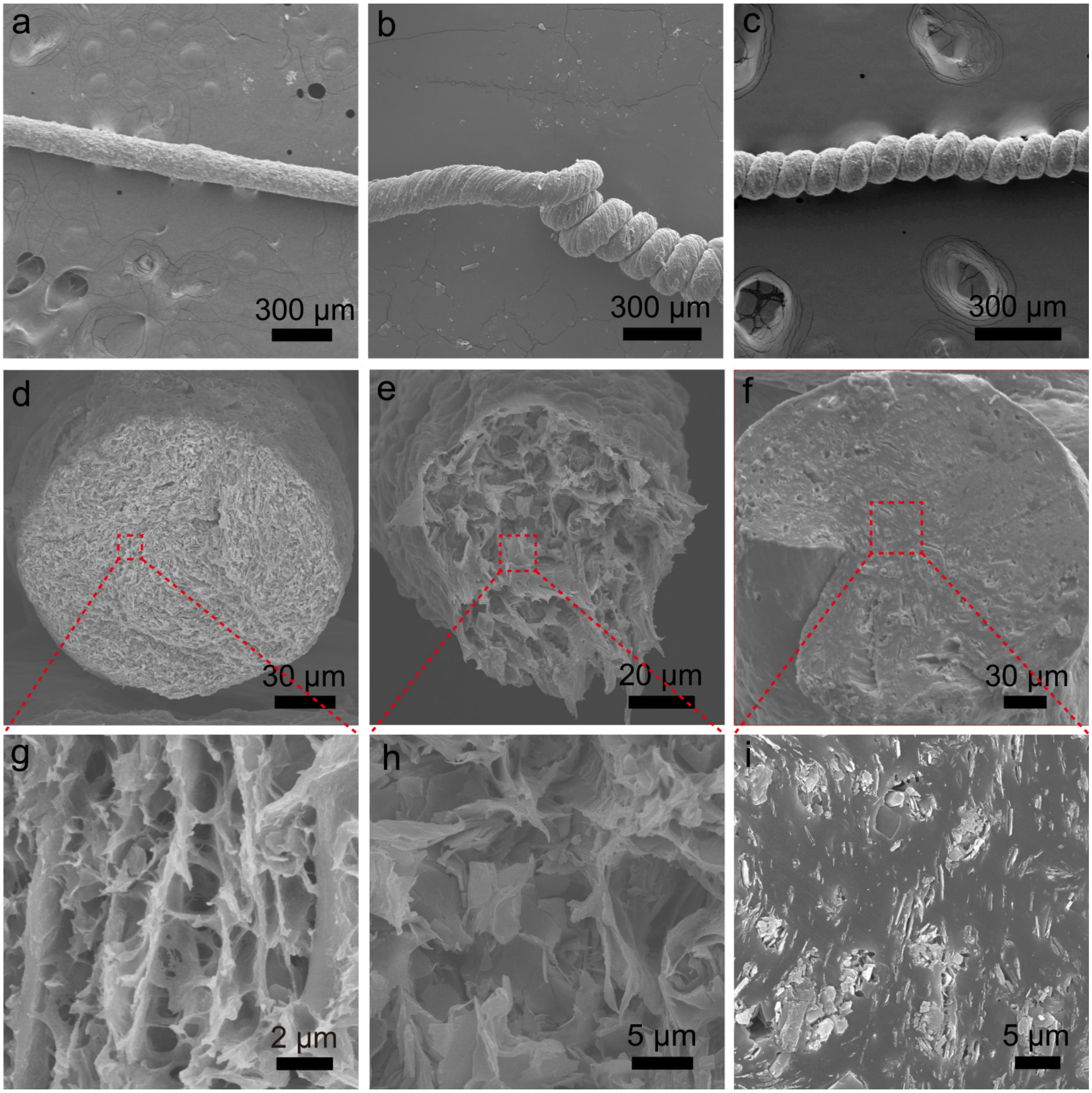

Supplementary Figure 5 Characterizations of the microscale morphology during spinning process, the PVA content is 67 wt%. The evolutions of PVA/$CaCO_3$ composite fibers with the morphologies of (**a**) composite fiber once drawing from the coagulation bath, (**b**) circular fiber with several loops after twist–spinning, (**c**) spring–like fibers formed by further twist–spinning, (**d–f**) SEM images of cross–section of the three types of fiber shown in Supplementary Fig. 5**a**, **b**, **c**. (**g–i**) SEM images of cross–section marked in red box in Supplementary Fig. 5**d–f**.

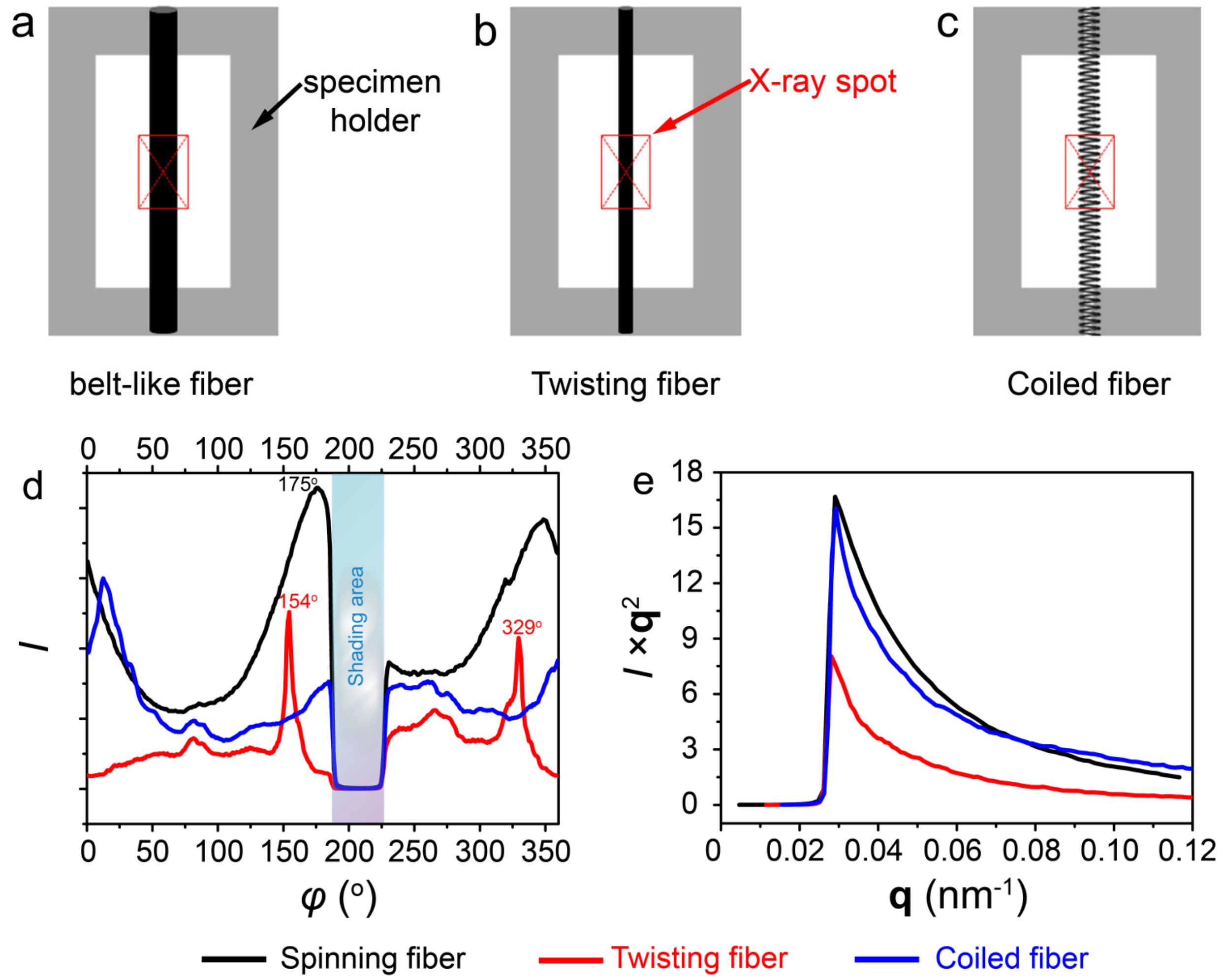

Supplementary Figure 6 Illustration of SAXS testing setup for three specimens of PVA/*G* fiber. (**a**) Belt–like fiber after wet–spinning, (**b**) Twist-spinned fiber before development of helical morphology, (**c**) Nacre–like fiber after formation of coils. The area of incident X–ray spot is 400 × 600 μm^2. All the specimens were vertically fixed, (**d**) Profile of scattering intensity (I) as a function of azimuthal angle (φ). (**e**) Profile of scattering intensity ($I \times \mathbf{q}^2$) as a function of scattering vector (**q**) ($\mathbf{q} = 4\pi\sin\theta/\lambda$).

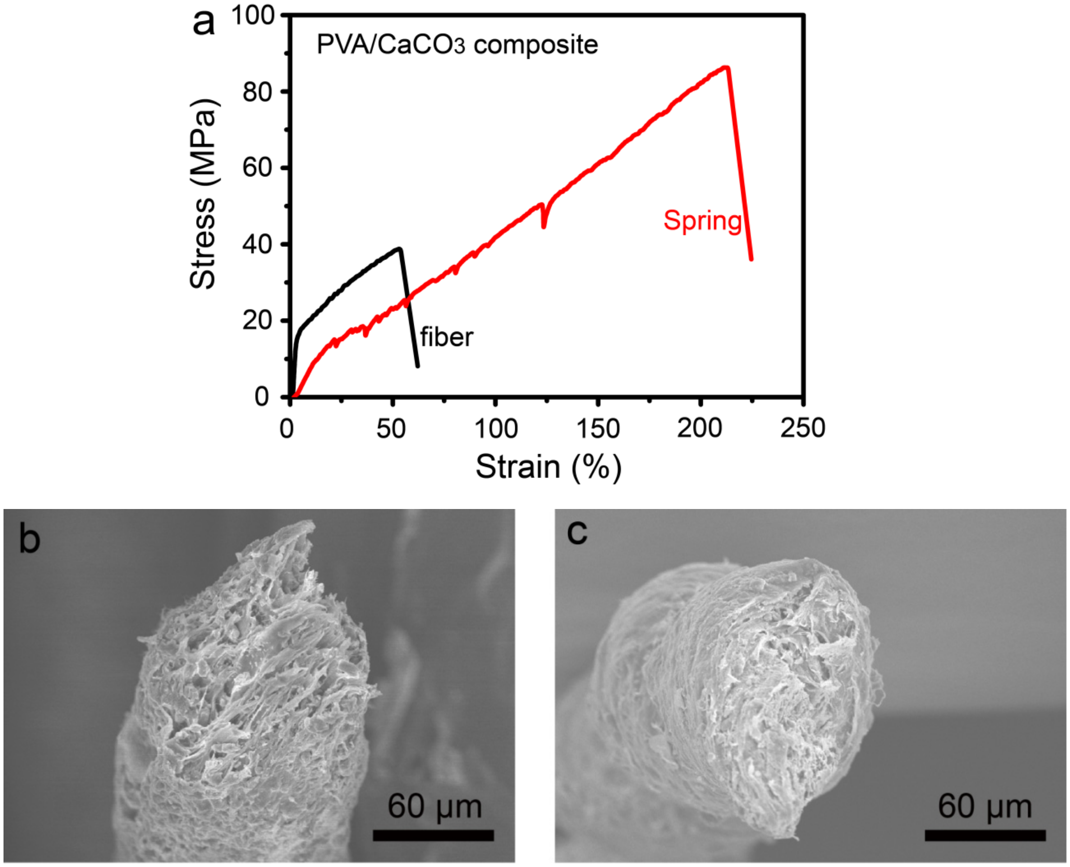

Supplementary Figure 7 Properties of PVA/$CaCO_3$ fibers. (**a**) Typical stress-strain curves of the early nacre-like ~67% PVA/$CaCO_3$ composite fiber and coiled fiber after twist-spinning step. The elongation, ultimate stress and toughness of the fiber are in the range of 52 ± 2%, 69 ± 4 Mpa, and 19.8 ± 2.2 J g^{-1} (density=1.57 g cm^{-3}), respectively. After twist-spinning, these parameters are 200.6 ± 14%, 84.8 ± 0.84 Mpa, and 107.1 ± 11.6 J g^{-1}, respectively, (**b**, **c**) Typical tensile fracture morphology SEM image of the belt-like fiber and coiled nacre-like fiber.

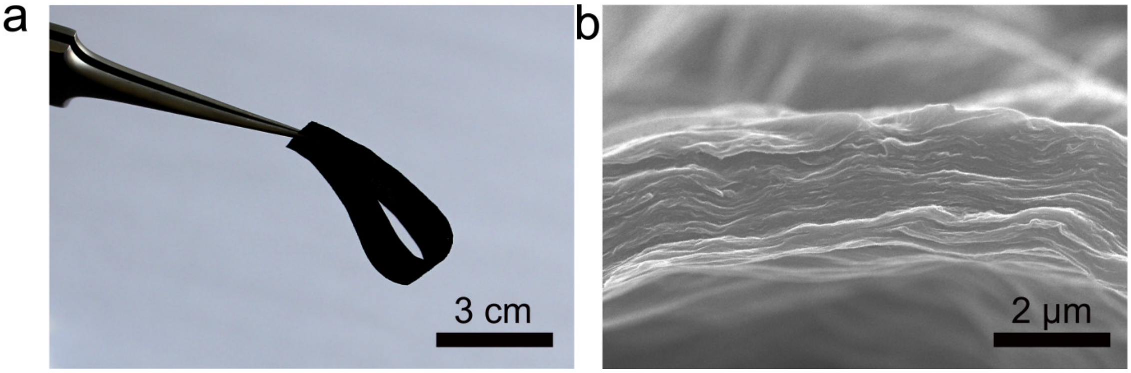

Supplementary Figure 8 Characterization of the ~66% PVA/*G* composite film prepared by vacuum assisted filtration (VAF). (**a**) Photography of the film, (**b**) SEM image of the cross–section of nacre–like film showing the typical brick–and–mortar structure.

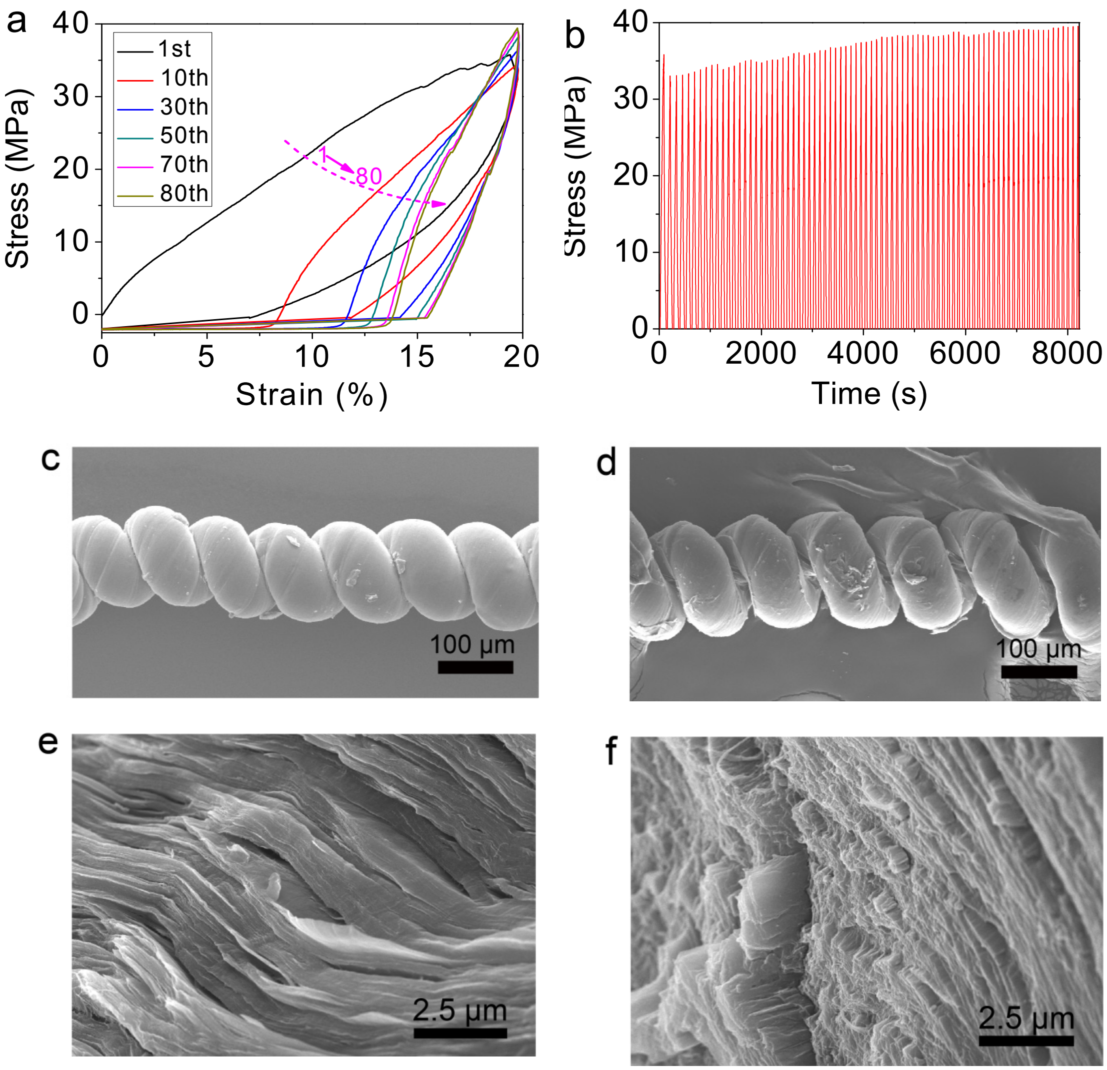

Supplementary Figure 9 The structural evolution and mechanical properties of nacre–like PVA/*G* fiber with deformation cycles. (**a**) Selected loading–unloading stress–strain curves for cycle 1st, 10th, 30th, 50th, 70th, and 80th with $\varepsilon = 20\%$, (**b**) Tensile stress as a function of time during cyclic testing, (**c**, **d**) SEM images of morphology of coiled nacre–like fiber before and after 80 cycle tensile tests, (**e**) SEM image of fracture morphology of the coiled nacre–like fiber, (**f**) SEM image of fracture morphology coiled nacre–like fiber after cyclic tensile tests.

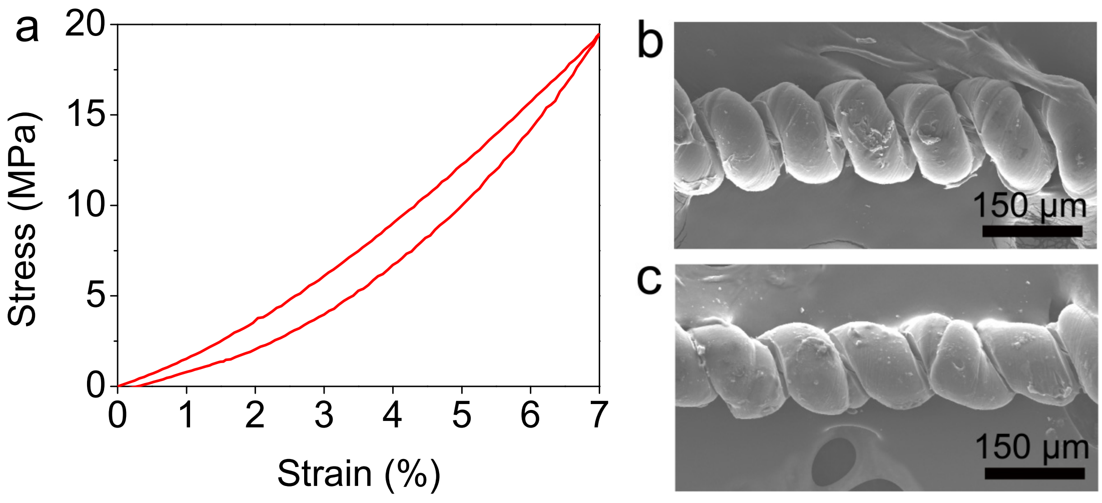

Supplementary Figure 10 The recovery properties of PVA/*G* coiled fiber after pre–stretched cycles. (**a**) Tensile stress–strain curve of coiled fiber at ε = 7% after 80 cycles stretching. The energy dissipation (~0.13 J g^{-1}) during shortening of the spring was confirmed by the visible hysteresis between the loading and unloading curve, which indicated a viscoelastic behavior of the nacre–like fiber. (**b**, **c**) SEM images of morphology of coiled fiber before and after stretching at ε = 7% after 80 cycles tensile test. The elastic spring constant (k) can be calculated by the equation: $k = Gd^4/64r^3N$, where G is shear modulus ($G = E/2(1+v)$), E is the elasticity modulus, v is Poisson's ratio (equal to 0.3), d is the overall fiber diameter (83 μm), r is the distance between the center of the loop and the yarn center along the radial direction (~30 μm), and N is the number of loops in the measured length (94 loops, partly shown in Supplementary Fig. 11a).

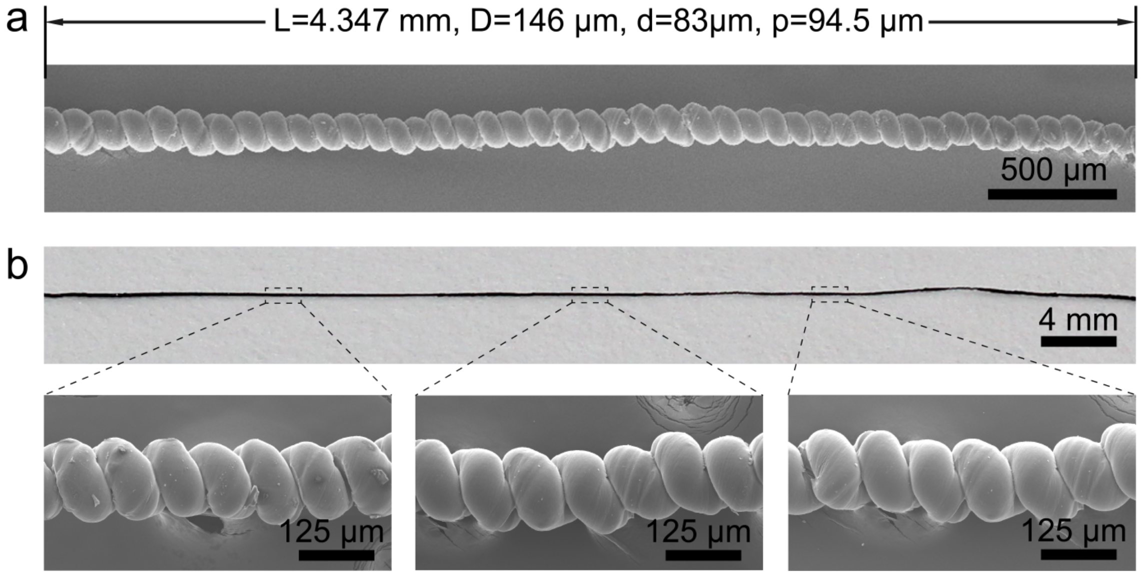

Supplementary Figure 11 Characterization of nacre–like PVA/*G* composite fiber with PVA fraction of 66 wt%. (**a**) SEM image of uniform the scaled–up version of the nacre–like PVA/*G* fiber, (**b**) Photograph of a 4 cm long PVA/*G* fiber in relaxed state, SEM images show the loops in different areas.

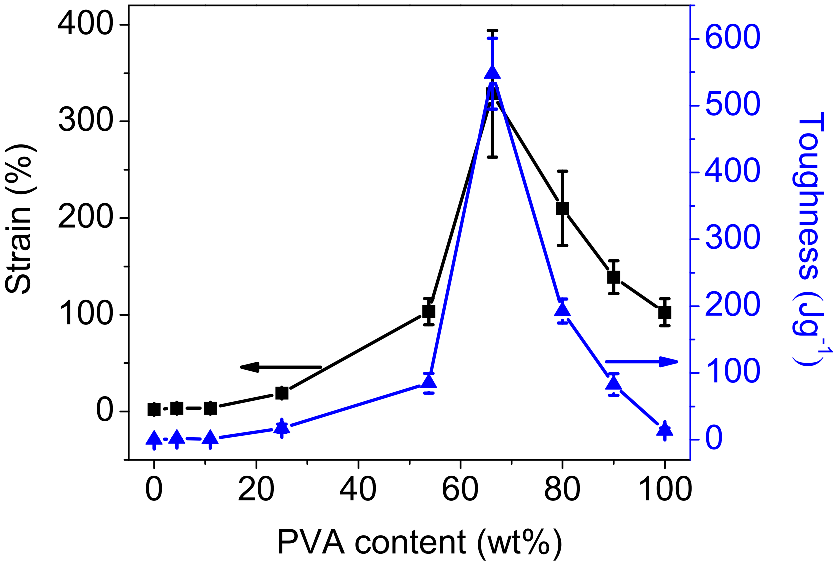

Supplementary Figure 12 The tensile strain (ε), toughness of the PVA/*G* fiber with different mass fractions of organic phase (PVA).

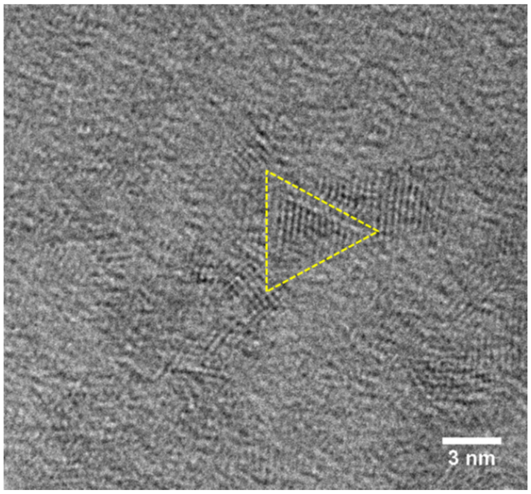

Supplementary Figure 13 TEM image of TGA–CdTe NPs. The yellow triangle highlights the tetrahedral shape of a CdTe NP with an approximate size of 3 nm. The lattice spacing is measured at 0.37 nm, corresponding to the <111> direction in zinc blende CdTe.

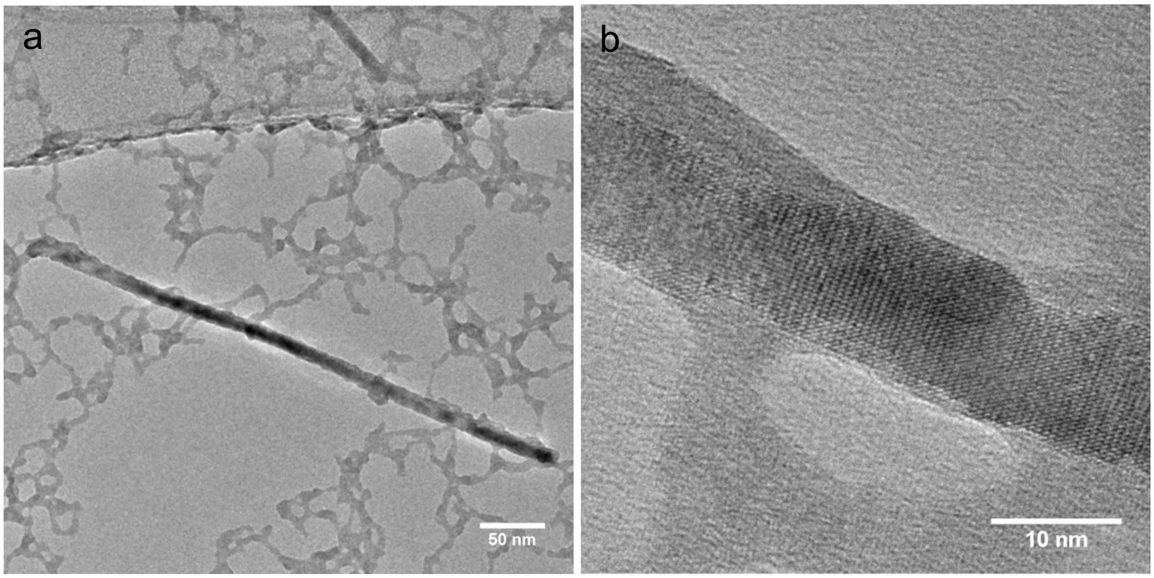

Supplementary Figure 14 TEM characterization of as–prepared TGA–CdTe nanowire. (**a**) Low magnification TEM image of TGA–CdTe nanowires with length of ~500 nm, (**b**) High–resolution TEM image of TGA–CdTe nanowires with width of ~10 nm.

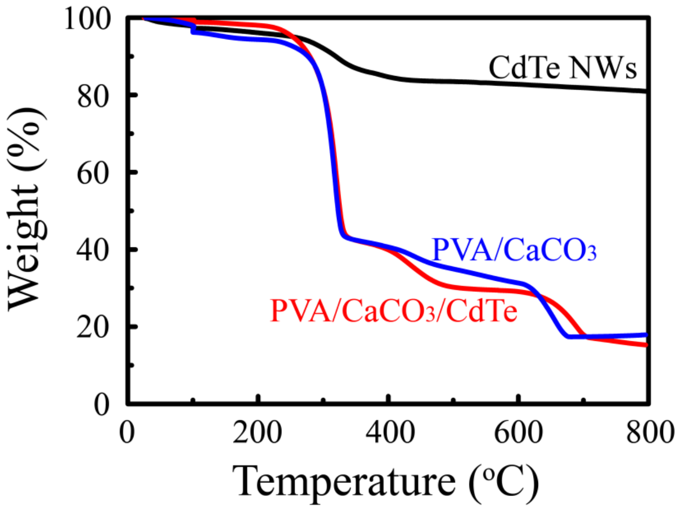

Supplementary Figure 15 TGA curves of the pure CdTe nanowire (black), PVA/$CaCO_3$ composite (blue) and PVA/$CaCO_3$/CdTe composite (red). The content of CdTe NWs in the PVA/$CaCO_3$/CdTe composite fiber was calculated to be about 0.9 wt%.

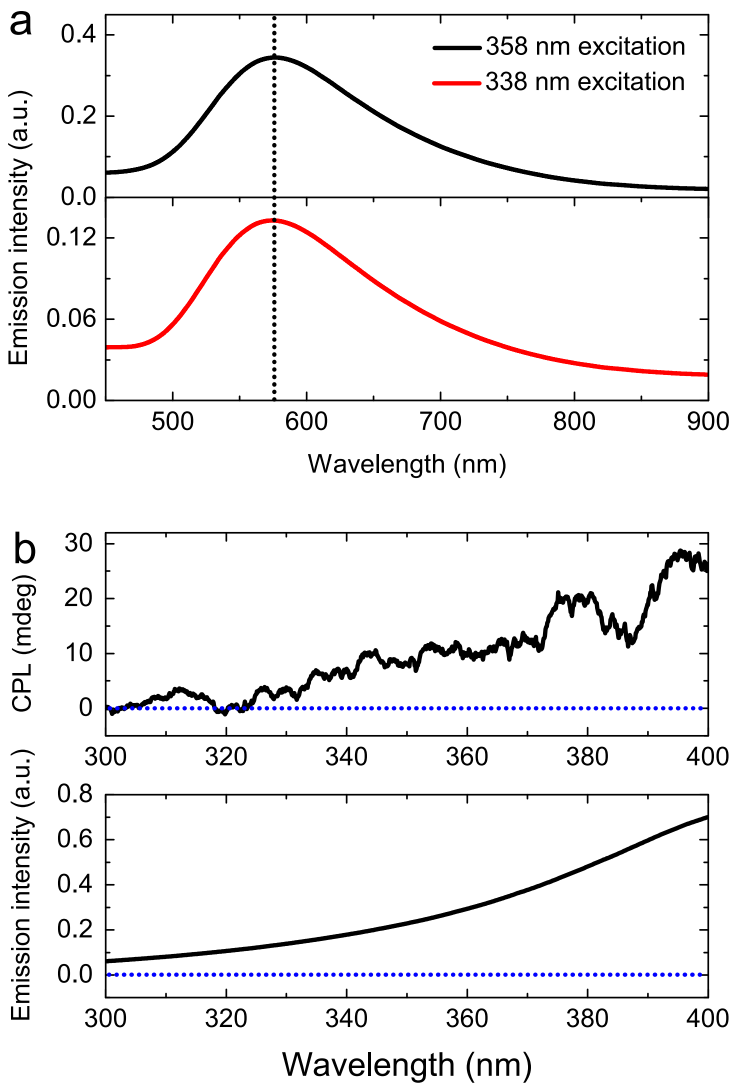

Supplementary Figure 16 (**a**) Emission intensity spectra of helical fibers containing CdTe NWs excited at 358 nm and 338 nm, respectively. The dotted line shows the luminescence maxima wavelength at 575 nm for both excitation wavelengths. The decrease in DC voltage for the 338 nm excitation compared to 358 nm excitation is due to the lower luminescence efficiency as well as a corresponding lower CPL value with a lower wavelength excitation, shown in (**b**).

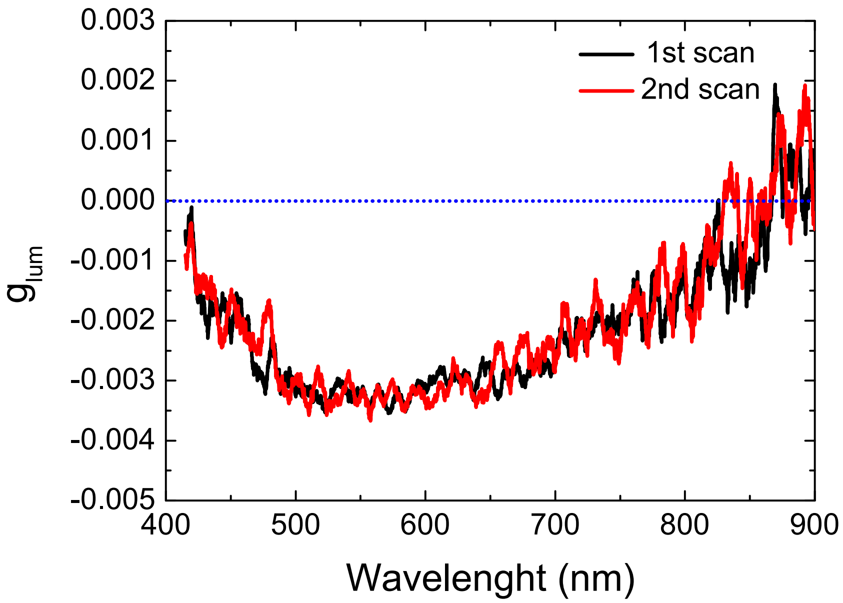

Supplementary Figure 17 g_{lum} spectra for two consecutive scans.

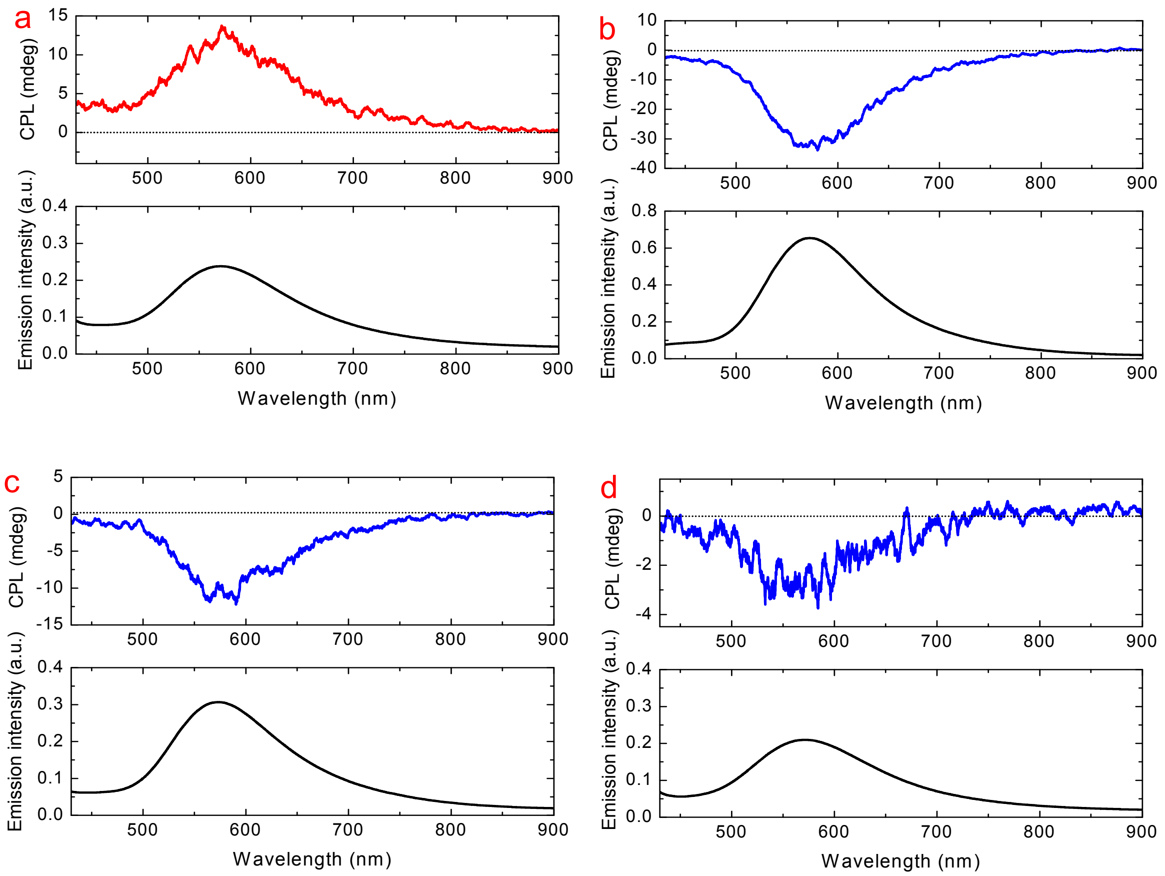

Supplementary Figure 18 CPL and emission intensity spectra of (**a**) left–handed fibers, and right–handed fibers stretched by (**b**) 0%, (**c**) 50%, and (**d**) 100%.

Supplementary Table 1 Comparison of mechanical properties of PVA/*G* fiber with the natural structure (nacre and spider silk), artificial fibers (nylon and kevlar), CNT– and graphene– based fibers

Fiber type	Strain (%)	Tensile strength (MPa)	Young's Modulus (GPa)	Toughness	Reference
Nacre (Pinctada)	2	80–135	60–70	1.8 MJ m^{-3}	1
Spider (Nephila edulis female)	39±8	1150±200	7.9±1.8	165±30 J g^{-1}	2
Kevlar (KM2)	4.52±0.37%	3880±400	84.62±4.18	36~78 J g^{-1}	3, 4
Nylon fiber	18	950		~80 J g^{-1}	5
CNT yarns	<13	150~300		11~20 J g^{-1}	6
CNT ropes	<285	73.8		28.7 J g^{-1}	7
CNT/PVA	<3	161	9.9		8
GO	6.8	102	5.4		9
GO/PVA	3	162	10	2.55 MJ m^{-3}	10
GO/HPG	1.6~5.6	72~158	7.8~15.9	18 MJ m^{-3}	11, 12
**PVA/*G***	**330±60**	**270 ±30**		**548.0±50 J g^{-1}**	**this work**

Supplementary References

1. Jackson, A. P. *et al*. The mechanical design of nacre. *Proc. R. Soc. Lond. B.* **234,** 415-440 (1988).
2. Vollrath, F. & Knight, D. P. Liquid crystalline spinning of spider silk. *Nature* **410,** 541-548 (2001).
3. Green, M. J., Behabtu, N., Pasquali, M. & Adams, W. W. Nanotubes as polymers. *Polymer* **50,** 4979-4997 (2009).
4. Cheng, M., Chen, W. & Weerasooriya, T. Mechanical properties of Kevlar®KM2 single fiber. *J. Eng. Mater. Technol.* **127,** 197-203 (2005).
5. Gosline, J. M., Guerette, P. A., Ortlepp, C. S. & Savage, K. N. The mechanical design of spider silks: from fibroin sequence to mechanical function. *J. Experi. Bio.* **202,** 3295-3303 (1999).
6. Zhang, M., Atkinson, K. R. & Baughman, R. H. Multifunctional carbon nanotube yarns by downsizing an ancient technology. *Science* **306,** 1358-1361 (2004).
7. Shang, Y. Y. *et al*. Super-stretchable spring-like carbon nanotube ropes. *Adv. Mater*. **24,** 2896-2900 (2012).
8. Dalton, A. B. *et al*. Super-tough carbon-nanotube fibres. *Nature* **423,** 703 (2003).
9. Xu, Z. & Gao, C. Graphene chiral liquid crystals and macroscopic assembled fibres. *Nature Commun*. **2,** 571-579 (2011).
10. Kou, L. & Gao, C. Bioinspired design and macroscopic assembly of poly(vinyl alcohol)-coated graphene into kilometers-long fibers. *Nanoscale* **5,** 4370-4378 (2013).
11. Hu, X. Z., Xu, Z. & Gao, C. Multifunctional, supramolecular, continuous artificial nacre

fibres. *Sci. Rep*. **2,** 767-774 (2012).

12. Hu, X. Z., Xu, Z., Liu, Z. & Gao, C. Liquid crystal self-templating approach to ultrastrong and tough biomimic composites. *Sci. Rep*. **3,** 2374-2381 (2013).
